# Encapsulated Papillary Oncocytic Neoplasm: A Newly Recognized Hurthle Cell Neoplasm With Unique Morphology

**DOI:** 10.7759/cureus.37175

**Published:** 2023-04-05

**Authors:** Noor Marji, Anwer Siddiqi, Arun Gopinath

**Affiliations:** 1 Pathology and Laboratory Medicine, University of Florida College of Medicine – Jacksonville, Jacksonville, USA

**Keywords:** epon, thyroid cytology, oncocytic neoplasm, hurthle cell neoplasm, encapsulated papillary oncocytic neoplasm

## Abstract

Diagnosis of oncocytic neoplasm of the thyroid gland can be challenging especially on fine needle aspiration biopsy (FNAB), given the wide differential diagnoses. In this report, we present the case of a 66-year-old male with an incidental thyroid nodule identified on imaging. In this case, identification of the distinctive cytologic features leads to the diagnosis of papillary oncocytic neoplasm on FNAB and helped to decide the appropriate surgical management. To date, the cytomorphologic features of this lesion are not well studied and established, with only a single case report in the literature. Herein, we describe the characteristic cytomorphologic and immunophenotypic features of this neoplasm. The recognition of the unique cytomorphologic features and awareness of the non-classic expression of the thyroid lineage markers will help in accurate diagnosis and management of this entity.

## Introduction

The term “oncocyte” or “oxyphil” is used to describe a thyroid follicular cell with ample granular eosinophilic cytoplasm due to the abundance of mitochondria. Oncocytic changes in the thyroid can occur as part of an inflammatory/immune-related response or as a part of the neoplastic process [1]. Once they become the predominant component and form an expansile growth, a diagnosis of oncocytic neoplasm should be considered. The 2022 World Health Organization (WHO) classification for endocrine and neuroendocrine tumors defines tumors of the thyroid with more than 75% oncocytic cells and absent papillary nuclear features as “oncocytic neoplasm” [2]. For follicular neoplasms, the presence of capsular or vascular invasion is considered a feature of aggressiveness. Oncocytic carcinomas constitute 3-5% of all differentiated thyroid carcinomas [3,4]. Ancillary studies like immunohistochemistry and molecular analysis provide minimal assistance in differentiating oncocytic tumor cells from other thyroid neoplastic follicular cell lesions. To date, the gold standard for diagnosis of thyroid lesions is histopathologic evaluation. The diagnosis of oncocytic lesions by fine needle aspiration biopsy (FNAB) is even more challenging [5]. We present this case of a rare morphologic variant of thyroid oncocytic neoplasm with papillary architecture, along with the description of its cyto-histomorphologic features.

## Case presentation

A 66-year-old male presented to the University of Florida, College of Medicine Jacksonville with a history of tobacco smoking, hepatitis C, liver cirrhosis, and thrombocytopenia with no family or personal history of radiation exposure, thyroid diseases, or autoimmune disease. CT scan revealed an incidental thyroid nodule and multiple bilateral lung nodules. PET CT showed an increased uptake in the thyroid gland, which was initially thought to represent a metastatic process from a lung primary.

**Figure 1:**
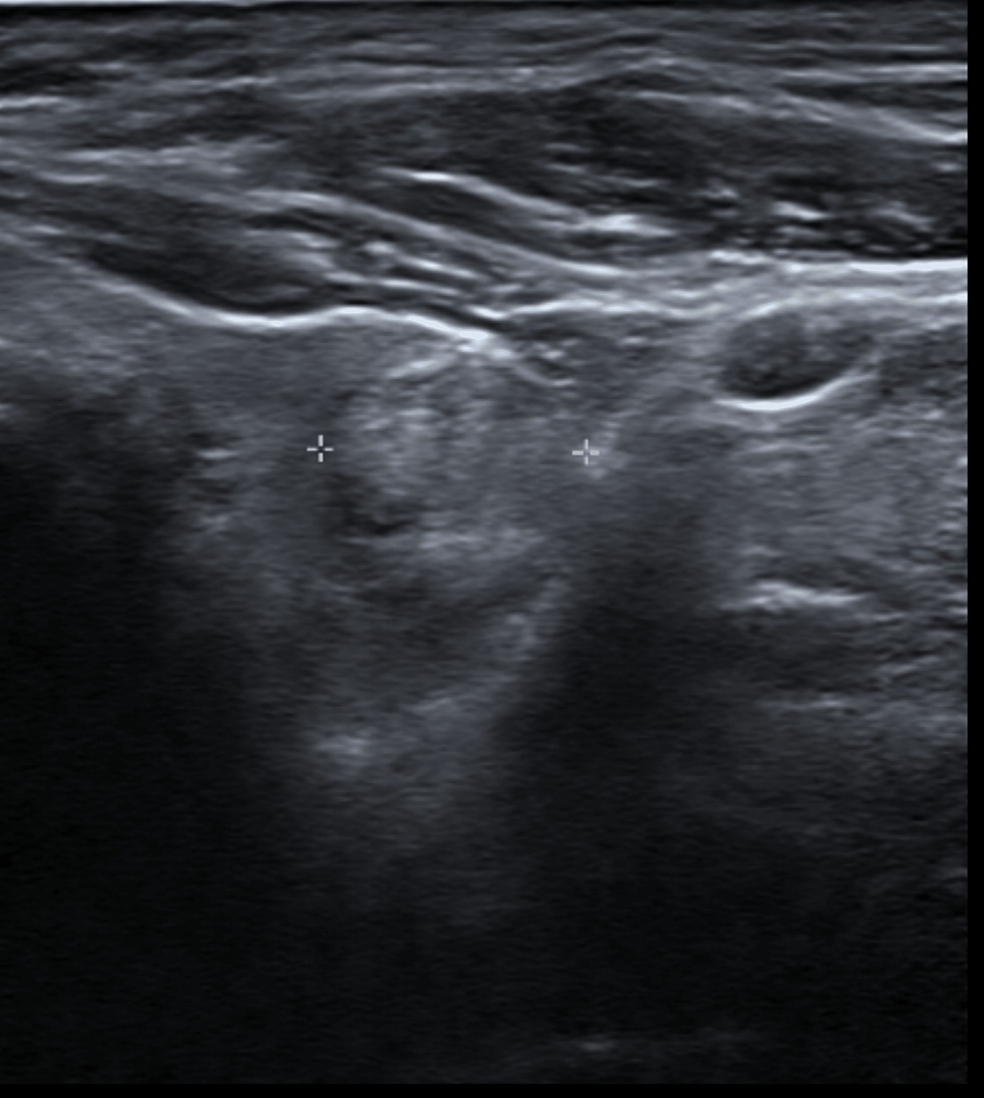
Lobulated isoechoic nodule in the upper pole of the left thyroid lobe measuring 1.1 x 1.5 cm.

Ultrasound of the thyroid gland was subsequently performed and confirmed a lobulated 1.5 cm isoechoic nodule in the upper pole of the left thyroid lobe (Figure 1).

**Figure 2:**
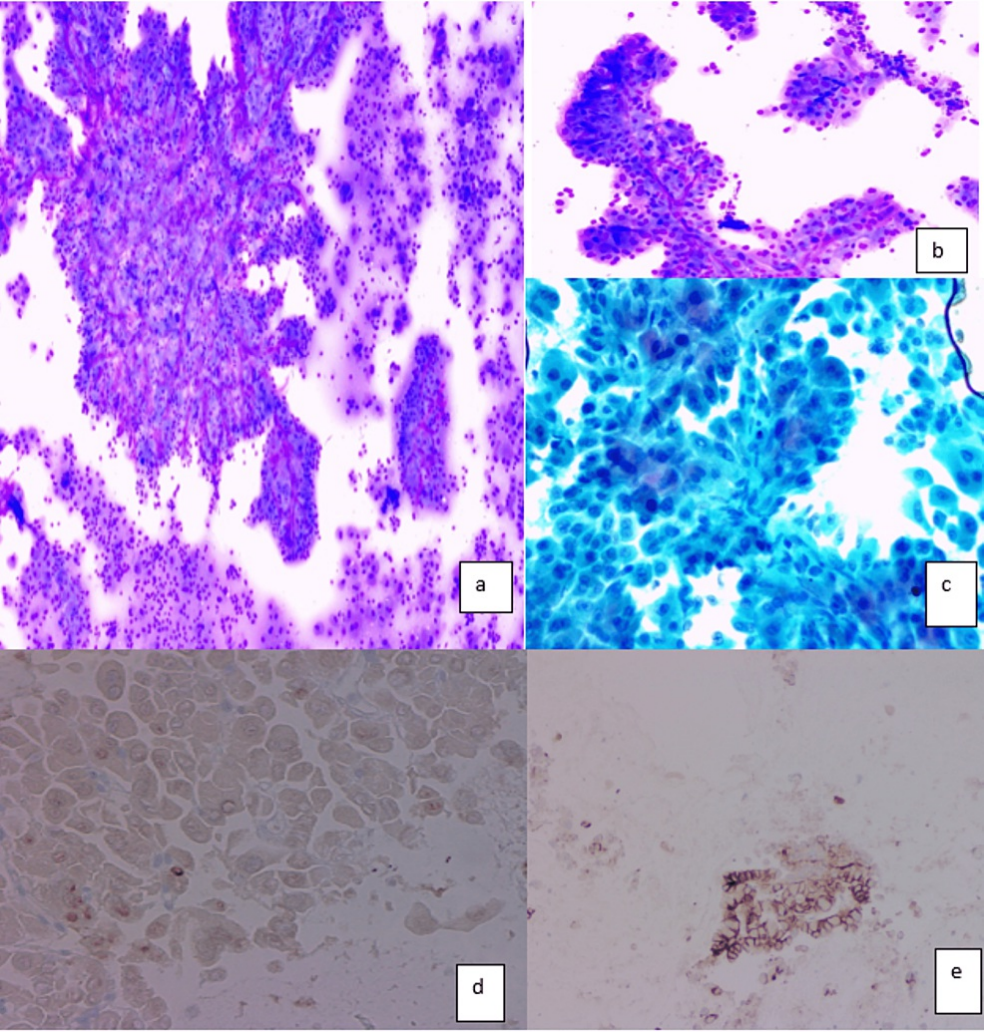
Thyroid FNA. (a and b) Smear showing prominent papillary architecture and oncocytic cells stained with Diff-Quik stain. (c) Pap stain. (d) PAX8 immunostain, staining few tumor cell nuclei. (e) Diffuse CD56 immunostaining.

Fine needle aspiration of the thyroid nodule was performed. Diff-Quik, Pap stain, and H&E cell block preparations were examined. Cytologic evaluation revealed a hypercellular specimen composed of polygonal cells with abundant eosinophilic cytoplasm and centrally placed nuclei, consistent with Hurthle (oncocytic) cells predominantly arranged in true papillary fibrovascular cores (Figures 2a-2c). On immunohistochemistry, tumor cells diffusely expressed CK7 and CD56 (Figure 2e). There was focal weak expression of thyroid lineage markers TTF-1 and PAX-8 (Figure 2d), and thyroglobulin was completely negative.

**Figure 3:**
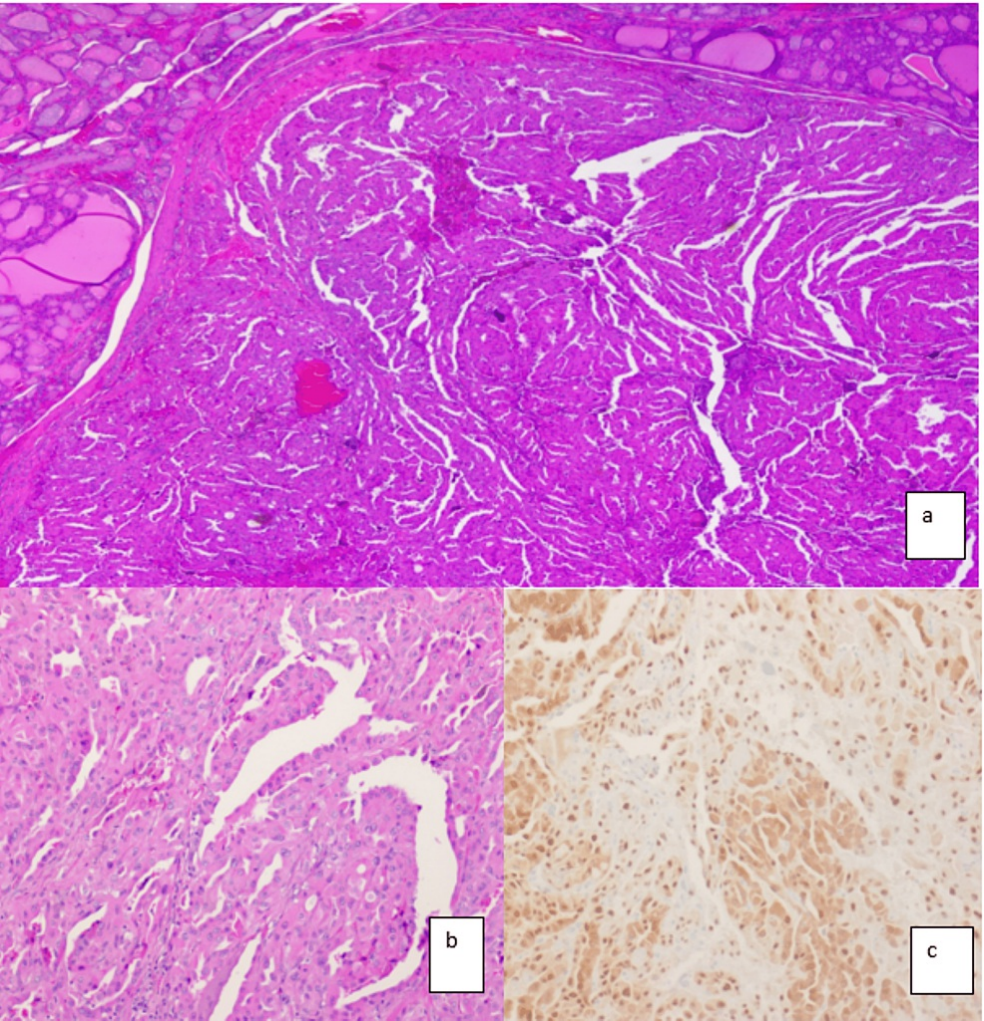
Encapsulated papillary oncocytic neoplasm (EPON). (a and b) Hurthle (oncocytic) cells predominantly in papillary/tubular configuration. (c) TTF-1 immunostain positive in tumor cell nuclei.

 Based on the above findings, possibilities of primary oncocytic neoplasm vs. metastatic oncocytic tumor were considered, and a conservative surgical excision was recommended. Left lobectomy was performed, and gross examination of the specimen revealed a 1.5 cm encapsulated tan-white nodule. On histologic evaluation of the nodule, it was entirely composed of Hurthle (oncoytic) cells in a predominant papillary configuration. Other architectural patterns including microfollicular and solid patterns were also noted (Figure 3). The papillary structures were composed of a single layer of cuboidal to columnar cells with an intervening hobnailing pattern. The nuclei were centrally located, with fine chromatin texture and small nucleoli. Minimal nuclear atypia including pleomorphism and nuclear membrane irregularity were noted. The capsule surrounding the nodule was entirely evaluated and did not show any evidence of capsular invasion. No vascular invasion was identified. In contrast with the cytology preparation, diffuse expression of both TTF-1 and PAX-8 was noted supporting thyroid origin of the nodule.

## Discussion

Thyroid oncocytic neoplasms are considered variants of follicular neoplasms. The 2017 WHO classification of thyroid tumors classifies Hurthle (oncocytic) cell tumors into Hurthle cell adenoma and Hurthle cell carcinoma [6]. As per the 2022 WHO new fifth edition classification of endocrine and neuroendocrine tumors, “oncocytic neoplasm” is the favored terminology for both Hurthle cell adenoma and carcinoma [2]. Oncocytic neoplasms can have different architectural patterns with the microfollicular variant being the most common, composed entirely/almost entirely of uniform follicles with scant colloid. Others include solid and/or trabecular patterns [7]. Focal papillary changes may be seen due to the inherent nature of these cells which can make them liable for fragmentation and falling apart during processing or fixation forming papillary structures which are more accurately referred to as pseudopapillary [1]. However, true papillary architecture can be rarely seen in oncocytic neoplasms. There is a paucity of literature on this entity [8,9,5]. Berho and Suster in their study on oncocytic papillary carcinoma of the thyroid suggested excluding the nuclear features of papillary carcinoma before diagnosing these tumors [10]. They recommended the “papillary variant of Hurthle cell tumor” nomenclature for these lesions. Even though morphology is the gold standard in this distinction, immunostains like CD56 have shown some promise. Lack of CD56 expression was shown to have high specificity and sensitivity for diagnosis of PTC [11]. Papillary thyroid carcinomas have molecular abnormalities that alter the MAP kinase signaling pathway by continuous activation including RET/PTC, BRAF, and RAS mutations [12]. Recently, Woodford et al. did molecular analysis of 18 cases of encapsulated papillary oncocytic neoplasms of the thyroid (EPONs), and none of them showed point mutation on BRAF mutation analysis which accounts for 70% of PTC cases [9]. In addition, the RET/PTC rearrangement appears to be negative in EPONs, supporting the concept that EPONs are different from PTC. Interestingly, FISH analysis did exhibit trisomy 10 in one case. As with follicular carcinomas, the criteria to classify oncocytic neoplasms being benign or malignant are based on the identification of capsular or vascular invasion identified on resection specimens.

Cytomorphologic features of this rare entity are not well studied. To the best of our knowledge, there is only one case report in the literature that describes the cytologic features of this entity on FNAB [5]. In our patient, the cytologic preparations (Diff-Quik and Pap=stained slides) showed prominent papillary configuration with abundant oncocytic cells, characterized by abundant eosinophilic granular cytoplasm, centrally located nuclei, and small nucleoli (Figure 2). There was only a mild increase in the nuclear size with no mitotic figures or necrosis. Papillary carcinoma nuclear features were entirely absent including (grooves, pseudoinclusions, clearing, irregular nuclear contours, and overlapping). The neoplastic cells were positive for Keratins and CD56, with focal expression of thyroid lineage markers (PAX8, TTF-1, and Thyroglobulin). The oncocytic and papillary nature, focal expression of thyroid lineage markers, and positive staining for PAX8 also raised the possibility of a metastatic process from the kidney-like papillary carcinoma. Even though PAX8 can be expressed in renal neoplasms, expression of TTF-1 and thyroglobulin would be a rare occurrence. The diffuse membranous staining of CD56 further supported the diagnosis of EPONs. In lobectomy specimens, the most important findings were the absence of PTC nuclear features (grooves, inclusions, clearing, and overlapping) and the absence of capsular and vascular invasion. Unlike the cytologic immunophenotype, there was diffuse expression of TTF-1 and PAX8 in the resection specimen, supporting the diagnosis. Table 1 shows the summary of the differential diagnosis and relevant immunostains/ molecular abnormalities.

**Table 1:** Summary of the differential diagnosis with relevant immunostain results and molecular abnormalities.

Differential diagnosis	PAX-8	TTF-1	Thyroglobulin	CD56	Molecular abnormalities
Encapsulated papillary oncocytic neoplasm (EPON)	+	+	+	+	May show trisomy 10 on FISH analysis
Papillary thyroid carcinoma	+	+	+	-	RET/PTC BRAF RAS
Metastatic renal cell carcinoma, papillary type	+	-	-	-	Heterogeneous, losses or gains of chromosomes 1, 3, 4, 5, 6, 8, 9, 10, 11, 15, 18 and 22

## Conclusions

In conclusion, EPONs constitute a rare morphologic variant of Hurthle cell neoplasms of the thyroid with a biologic behavior like follicular tumors. The knowledge of this complex morphology as well as the non-characteristic immunophenotypic expression especially while evaluating cytologic preparations of oncocytic thyroid lesions will prevent misdiagnosis. Clinical and imaging correlation is extremely helpful in these cases, and also conservative excision of these lesions cannot be overemphasized.
